# Supplementary material for: Identifying training needs of healthcare providers to implement caries risk assessment
Source: Front Oral Health. 2025 Aug 29;6:1641307. doi: 10.3389/froh.2025.1641307 (PMC12425963; doi:10.3389/froh.2025.1641307)
Supplement: Supplementary file 1 [file Datasheet1.docx]

**Study Title: Exploring strategies for implementing a culturally informed caries risk assessment tool used by non-dental primary care providers for young First Nations and Metis children in Manitoba, Canada**

**FOCUS GROUP AND IN-DEPTH INTERVIEW GUIDE**

1. What are your thoughts on early childhood tooth decay?

- What comes to mind? What do you know about it?
- Do you think dental decay in children is preventable?

1. Tell me what you know about caries risk assessment (CRA)

- Have you used it or seen it being used?

1. How do you feel about non-dental primary care providers (nurses, physicians, dieticians, etc.) doing CRA for preschoolers?
2. Looking at CRA tool developed for use in Canada for preschool children, how do you foresee integrating it into your practice?
3. What are some benefits of integrating CRA for preschoolers into your practice?
   - For you, for your patient, for your clinic?
4. What challenges/barriers do you anticipate?
   - For you, for your patient, for your clinic?
5. Have you already implemented any form of CRA into your practice?

- If yes, please describe your experience.
- If not, what will help you successfully implement CRA in your practice?

1. What training do you like or think you need to implement CRA successfully?

- What will be the best way to implement the training?

1. The CRA tool may prompt you to apply fluoride vanish. How do you feel about it?

- What challenges/barriers do you anticipate in your practice?

1. In your opinion who do you see as an ideal person(s) within your practice to champion CRA implementation
2. What challenges do you anticipate encountering when connecting/referring to dental providers?
3. What challenges do you anticipate encountering when giving preventive advice?
4. Will you be using the CRA tool in your practice?

- If not, why?

1. Please share any other thoughts or comments on using the tool itself.
